# Co-Ingestion of Natal Plums (*Carissa macrocarpa*) and Marula Nuts (*Sclerocarya birrea*) in a Snack Bar and Its Effect on Phenolic Compounds and Bioactivities

**DOI:** 10.3390/molecules27010310

**Published:** 2022-01-04

**Authors:** Vimbainashe E. Manhivi, Retha M. Slabbert, Dharini Sivakumar

**Affiliations:** 1 Phytochemical Food Network Group, Department of Crop Sciences, Tshwane University of Technology, Pretoria West 0001, South Africa; ManhiviVE@tut.ac.za; 2 Department of Horticulture, Tshwane University of Technology, Pretoria West 0001, South Africa; SlabbertMM@tut.ac.zam

**Keywords:** bioaccessibility, indigenous food, antioxidant properties, phenolic compounds, anthocyanins, gastrointestinal digestion

## Abstract

This study investigated the effect of co-ingesting Natal plums (*Carissa macrocarpa*) and Marula nuts (*Sclerocarya birrea*) on the bioaccessibility and uptake of anthocyanins, antioxidant capacity, and the ability to inhibit α-glucosidase. A Natal plum–Marula nut bar was made by mixing the raw nuts and the fruit pulp in a ratio 1:1 (*v*/*v*). The cyanidin-3-*O*-sambubioside (Cy-3-Sa) and cyanidin-3-*O*-glucoside content (Cy-3-G) were quantified using the ultra-high performance liquid chromatography-quadrupole time-of-flight mass spectrometry (UHPLC/Q-TOF-MS). Inclusion of Natal plum in the Marula nut bar increased the Cy-3-Sa, Cy-3-G content, antioxidants capacity and α-glucosidase inhibition compared to ingesting Marula nut separately at the internal phase. Adding Natal plum to the Marula nut bar increased bioaccessibility of Cy-3-Sa, Cy-3-G, quercetin, coumaric acid, syringic acid and ferulic acid to 80.2% and 71.9%, 98.7%, 95.2%, 51.9% and 89.3%, respectively, compared to ingesting the Natal plum fruit or nut separately.

## 1. Introduction

Natal plum (*Carissa macrocarpa*) and Marula (*Sclerocarya birrea)* fruits are popular among the rural people from southern Africa. Anthocyanins (cyanidin-3-*O*-β-sambubioside, cyanidin-3-*O*-glucoside) have been identified as the major phenolic compound in the Natal plum [1]. Anthocyanins are polyphenolic compounds that belong to the flavonoid family [2]. Besides giving berries, fruits, and certain vegetables-based products their red–blue color, anthocyanins also have health-promoting properties [3]. In the United States, anthocyanin intake is estimated at 12.50 mg per day [4]. Foods containing anthocyanins and anthocyanin-rich compounds have been shown to display several biological activities with potentially positive health implications. These include anti-inflammatory, anti-diabetic, and antioxidant activities [3]. While anthocyanins can protect consumers against chronic diseases, they are susceptible to nume rous factors, including their bioavailability: namely, their absorption, metabolism, distribution, and excretion [3]. In our previous studies, we found that Natal plum showed strong inhibitory properties against carbohydrate-hydrolyzing enzymes, particularly α-glucosidase due to its anthocyanin content [5]. However, antioxidant capacity and α-glucosidase inhibition decreased during simulated gastrointestinal digestion [6]. Since anthocyanins are water-soluble contents of the vacuole, they are easily released during digestion [7]. In addition, anthocyanins are unstable in the near-alkaline conditions of the intestine, where they rapidly degrade or form other phenolics, which might have lower antioxidant and anti-diabetic properties [8]. The bioaccessibility of anthocyanins is dependent on their structure. The acylation of anthocyanins increases their stability and substantially reduces their bioavailability [8,9]. Pelagonidin-based anthocyanins (3′-hydroxyanthocyanins) are more readily absorbed than anthocyanins with more substituents on the B-ring [10]. Based on the chemical structure of the anthocyanidin backbone, the degree of glycosylation and hydroxylation of flavylium molecules, as well as the number, type, and acylation of bound sugar molecules, are directly related to plant species. Therefore, the plant species and food matrix (fruit components) play a significant role in determining the content, bioaccessibility, and bioavailability of anthocyanins. In some cases, phenolic compounds may form covalent or non-covalent bonds with fibres, proteins, and carbohydrates that affect their release [11]. In addition, this interaction may take place prior to harvest in the fruit cells or when the fruit is co-digested with other foods [12]. Pomegranate anthocyanins’ bioaccessibility was influenced by food macromolecules such as sugar, starch, and cellulose [13]. Bioaccessibility of Natal plum phenolics and anthocyanins has been assessed assuming that there is no interference of other foods during digestion [5,6]. The concentration of Natal plum phenolics and anthocyanins was seen to decrease during simulated gastrointestinal digestion. It is, however, realistic to consider the fact that fruits are usually consumed with other fruits or nuts in the form of pestils, with peanuts and raisins, or in snack bars.

Food components ingested with anthocyanins can increase or decrease their bioaccessibility depending on a number of factors, including the type of anthocyanin, the degree and type of food processing, including pH, temperature, light conditions, as well as metal ions or oxygen [14]. An increase in the bioaccessibility of anthocyanins and carotenoids was observed when red cabbage was co-digested with different carotenoid-rich vegetables [15]. Human studies showed no effect of blackcurrant juice or rice cake on peak plasma anthocyanin concentration or absorption or excretion of anthocyanins, providing evidence that prolonged stay of rice cake in the stomach slowed the intestinal absorption of anthocyanins [16]. McGougall et al. [17] showed, in an in vitro digestion study on raspberry anthocyanins’ bioavailability, that anthocyanins were stable to gastric conditions, but poorly available after pancreatic digestion; the bioavailability was not affected by the intake of other complex food products. Gui-Fang et al. [18] found that the combination of fruits caused different interactions along the gastrointestinal tract that influenced the phenolic content recovered after digestion, suggesting that when various types of fruits are consumed together, their antioxidant capacity may differ from what would be expected from examining their individual antioxidant capacities. The presence of strawberries in the food matrix facilitated the bioavailability of cyanidin-based carrot anthocyanins during co-ingestion of black carrot and strawberry [2]. In contrast, Ribnicky et al. [19] showed, using their gastrointestinal model (TIM-1), that lipid-rich matrices did not affect anthocyanin bioavailability, while protein-rich matrices protected anthocyanins during transit through the upper digestive tract. Phan et al. [15] demonstrated, on the other hand, that dietary lipids protect black carrot anthocyanins and phenolics.

The pulp of Marula nuts, a waste product and rich source of protein and fat content [20], was used to produce a Natal plum–Marula fruit bar in this study. Utilising the nuts discarded from Marula fruit could improve revenue for farmers and increase dietary diversity. Moreover, the high protein and fat content in the nuts may enhance the bioaccessibility of Natal plum anthocyanins. Furthermore, protein-bound phenolics were protected from the stomach’s adverse effects and became more bioaccessible [21]. The authors of these recent studies propose that during digestion, there is an interaction among the constituents of different food sources, resulting in the altered bioaccessibility of phytochemicals.

In light of this, it would be interesting to determine whether or not the consumption of Natal plums and a nut such as Marula nuts in the form of snack bars to improve the bioaccessibility of phenolics and anthocyanins. A snack bar would be a convenient way to encourage consumption of the fruit and nut. In this study, we investigated how the bioaccessibility of of cyanidin-3-*O*-*β*-sambubioside, cyanidin-3-*O*-glucoside, other polyphenols, antioxidant activities and α-glucosidase activity after the co-ingestion of Natal plum and Marula nut in a bar using an in vitro simulated gastrointestinal digestion model.

## 2. Results and Discussion

### 2.1. Identification of Anthocyanins in Natal Plum and Natal Plum–Marula Nut Bar

**Figure 1:**
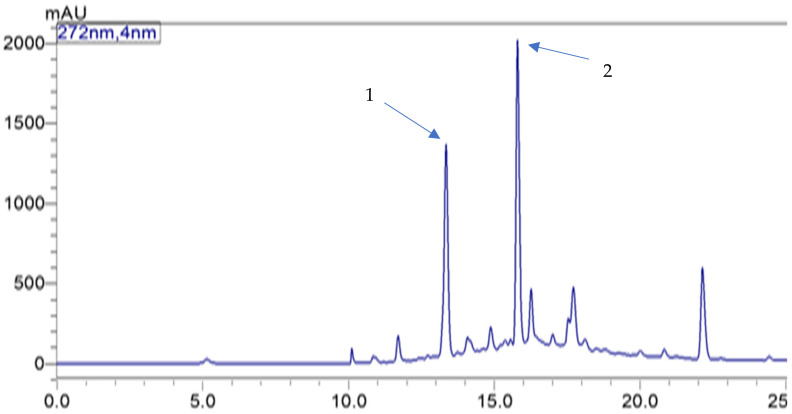
Chromatogram of phenolic compounds in the Natal plum–Marula nut bar. Major peaks 1 and 2 represent cyanidin 3-*O*-glucoside and cyanidin 3-*O*-sambubioside, respectively.

The MS data for the anthocyanin identification is presented in Supplementary Table S1. The tentative identification was based on the *m*/*z* as well as the fragmentation pattern as reported previously [5]. The peak tentatively identified as cyanidin-3-*O*-sambubioside had an observed *m*/*z* of 579.1 [M-2H]^−^ and an expected *m*/*z* of 581.5 possibly due to the loss of 2H^+^ from the parent ion. The peak tentatively identified as cyanidin-3-*O*-glucoside had an observed and expected *m*/*z* of 449.1 (Figure 1).

### 2.2. Effect of Enrichment and Co-Ingestion on Total Phenolic Content

**Figure 2:**
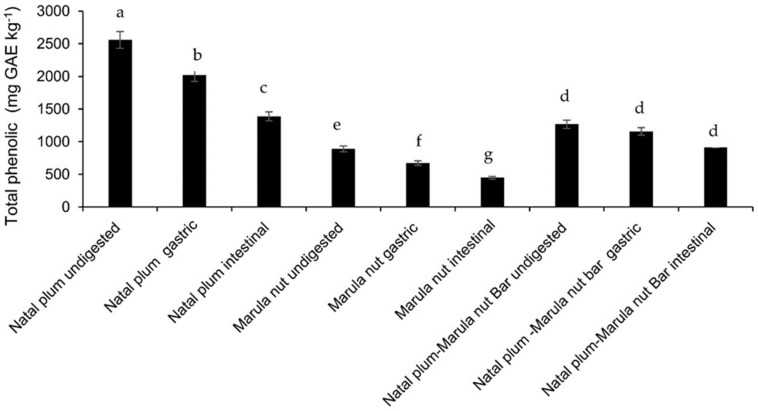
Effect of co-ingestion of Natal plum and Marula nut in a bar on the total phenolic content Means followed by the same letter in a bar are not significantly different at *p* < 0.05.

The total phenolic contents (TPC) of Natal plum, Marula nuts, and the bar, throughout the simulated gastrointestinal digestion phases are shown in Figure 2. Raw Marula nuts had the least TPC, whilst raw Natal plum had the highest. The high protein content of nuts may have caused most phenolic compounds to be bound, and this may hinder their extraction. Phenolics interact with carbohydrates and proteins through covalent and non-covalent bonds influence their extraction [2]. However, Natal plums’ major phenolic components are anthocyanins, which can be readily extracted with solvents upon rupture of the cells [6]. Supplementing Natal plum with the Marula nuts reduced the TPC of the Natal plum-Manula nut bar significantly (*p* < 0.05) since Marula nuts were lower in phenolic content. Each stage of simulated digestion significantly (*p* < 0.05) reduced the TPC of Natal plum and Marula nut. In both Natal plum and the Marula nut, however, the TPC reduction in the intestinal phase was significantly greater than that in the gastric phase. The decrease in TPC during simulated digestion may be attributed to the degradation of phenolics after aglycone formation in the gastric phase or due to reduced stability in the alkaline to neutral pH of the small intestine. Similar observations have also been reported for the Natal plum by Seke et al. [5]. Natal Plum–Marula bars showed higher TPC levels in undigested, gastric, and intestinal phases in comparison to Marula nuts. The amount of TPC present in Natal plum–Marula nut bar at the intestinal phase was almost similar to the levels present in undigested Marula nut. TPC levels in the intestinal phase of Natal Plum–Marula nut bars were almost similar to those of the undigested Marula nuts. This showed that the phenolics in the Natal plum–Marula nut bar remained more stable during digestion. Dietary fiber in fruits may also bind to phenolics and prevent their release until bacterial enzymes break them down in the colon [18].

### 2.3. Changes in Bioaccessibility of Anthocyanins and Other Polyphenols after Simulated Co-Digestion of Natal Plum and Marula Nut

Natal plums were the only source of anthocyanins, cyanidin-3-*O*-sambubioside (Cy-3-Sa) and cyanidin-3-*O*-glucoside (Cy-3-G), found in the bar (Table 1). Cy-3-G and Cy-3-Sa from the Natal plum varied between 8.96% and 34.41% in the gastric phase. In contrast, the anthocyanin content of black carrot varied significantly with gastric conditions from 59% to 86% [2]. In the gastric phase, the stability of cyanidin derivatives depended on the type of cyanidin. Stability was highest for the ferulic acid derivative, and lowest for the non-acylated cyanidin derivatives. In contrast to nonacylated anthocyanins, acylated anthocyanins have been reported to be more stable to temperature and pH changes [17], even though the type of acylated group may also influence anthocyanin stability during digestion [22]. Nonetheless, it was found that the Natal plum ingested with Marula nut in the fruit–nut bar exhibited stability of Cy-3-Sa and Cy-3-G in the gastric phase by 15.28% and 7.07%, respectively (Table 2). It has previously been reported that food matrix influences anthocyanin bioaccessibility in vitro following food co-digestion. The bioaccessibility of anthocyanins in meat, soymilk, and cream was reduced when these foods were consumed with pomegranate juice [13]. According to our study, when Natal plum was consumed alone, the bioaccessibility of Cy-3-Sa and Cy-3-G was 39.7% and 32.8%, respectively. The bioaccessibility of Cy-3-Sa and Cy-3-G increased by 80.2% and 71.9%, respectively, when Natal plums and Marula nuts were consumed together as a bar. It is possible that the increased bioaccessibility of Cy-3-Sa and Cy-3-G is due to the protective effect of lipids and proteins, reducing the degradation of anthocyanins. Consumption of black carrots and strawberries also increased the cyanidin-based anthocyanin bioaccessibility [2]. Moreover, anthocyanins possess chelating abilities, which allow them to form stable complexes with metal ions, which can withstand gastric/intestinal digestion, although these complexes affect the recovery of metal ions depending on the structure of the anthocyanins [23]. Whether remaining cations present in the extracts could be the cause of the increases observed should be a matter of further research [2].

Natal plum and Marula nut are both highly bioaccessible sources of gallic acid, epicatechin, catechin, caffeic acid, vanillic acid, and ellagic acid when taken together in a fruit–nut bar. The increase in gallic acid and ellagic acid after intestinal digestion may be due to the presence of ellagitannins in Marula nuts. Polyphenols that are classified as ellagitannins usually contain at least one hexahydroxydiphenoyl moiety attached to a sugar molecule [24]. Upon hydrolysis of ellagitannins with acids or bases, ester bonds are hydrolyzed, causing the hexahydroxydiphenoyl group to spontaneously rearrange into ellagic acid [25]. Gallic acid is a dimeric derivative of ellagic acid [26]. A similar increase in ellagic acid was observed with a simulated digestion of pomegranate and was attributed to ellagitannin degradation as well as increased release from the food matrix [27]. It is possible that the increase in caffeic acid after intestinal digestion of Natal plums and Marula nuts together in a fruit and nut bar is due to its release from proteins or carbohydrates, as well as ease of extraction after digestion and reduced degradation. Most phenolic compounds form covalent bonds with proteins and carbohydrates, and these phenolics may be released after digestion [28]. Furthermore, when the Natal plum and Marula nut were co-ingested in a fruit bar, the bioaccessibility of other targeted phenolic compounds, such as protochatechuic acid, epicatechin, caffeic acid, vanillic acid, syringic acid, ellagic acid, quercetin, and coumaric acid, improved significantly. This may be due to the reduction in phenolic acid degradation in the small intestine and the increase in their extractability, possibly working synergistically. Grape extract synergistically improved the bioaccessibility of blueberry anthocyanins [28]. As previously shown by Seke et al. [5], some anthocyanin degradation products, such as protochatechuic acid and other phenolics that may have been more extractible due to digestion, may also further degrade in the small intestine under unfavourable pH conditions When consumed as a fruit and nut bar, the phenols released may have been protected from an unfavourable pH by lipids or proteins. Dietary lipids enhanced the bioaccessibility of anthocyanins and phenolics in black carrots [21]. Phenolics may also bind to proteins and be released during intestinal digestion, thereby reducing the amount of time the phenolic is in contact with digestion solutions and therefore protecting pH-sensitive compounds. During digestion, whey proteins bound to oat phenolics provided protection [19]. The bioaccessibility of black carrot anthocyanins and phenolics was improved by dietary lipids [21]. 

### 2.4. Effect of Enrichment and Co-Ingestion on Antioxidant Properties

**Figure 3:**
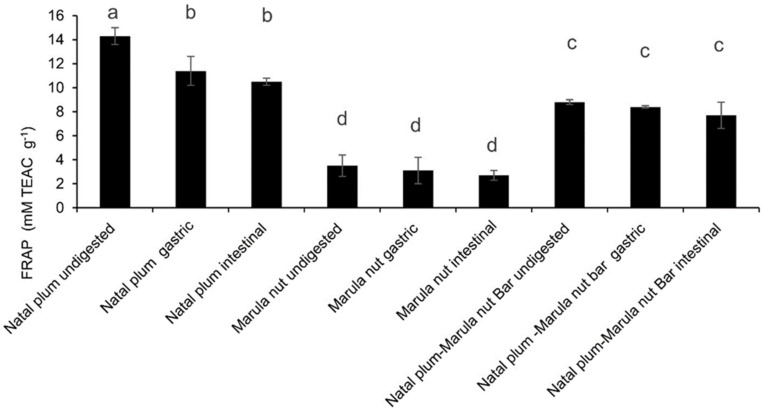
Effect of co-ingestion of Natal plum and Marula nut in a bar on the antioxidant power Means followed by the same letter in a bar are not significantly different at *p* < 0.05.

The effect of co-ingestion and simulated digestion on the antioxidant capacity of the Natal plum–Marula bar was also studied and compared to consumption of the nut or fruit alone. A reduction in Ferric Reducing Antioxidant Power (FRAP) was observed when Natal plums were combined with Marula nuts in the bar, probably because the nuts have lower antioxidant power (Figure 3). The antioxidant activity of the digesta of the gastrointestinal phase of the Natal plum, however, was higher than the Natal Plum–Marula fruit bar, followed by the Marula nuts. Although the reduction in antioxidant power may be due to phenolic degradation in the gastric phase of digestion, the non-significant decrease that follows may reflect the production of more potent antioxidants after the intestinal phase due to degradation of those already present or increase in the extractability of phenolics. The TPC of the Natal plum decreased during intestinal digestion, while the antioxidant power did not significantly differ. This shows that only one antioxidant activity determination method is not sufficient due to various shortcomings. It has been reported that Folin–Ciocalteu assays are interfered with by amino acids and purines released from proteins and nucleic acids as a result of enzymatic catalysis during digestion [29,30]. The insignificant change in antioxidant power also observed after digestion of Marula nuts and the bar may be due to the formation of other hydrolysis products such as peptides, which may have antioxidant activities. In studies, peptides produced during protein hydrolysis increased the antioxidant potency of green gram, horse gram, lentil, chickpeas, cowpeas, black peas, and white peas after simulated digestion [30,31].

**Figure 4:**
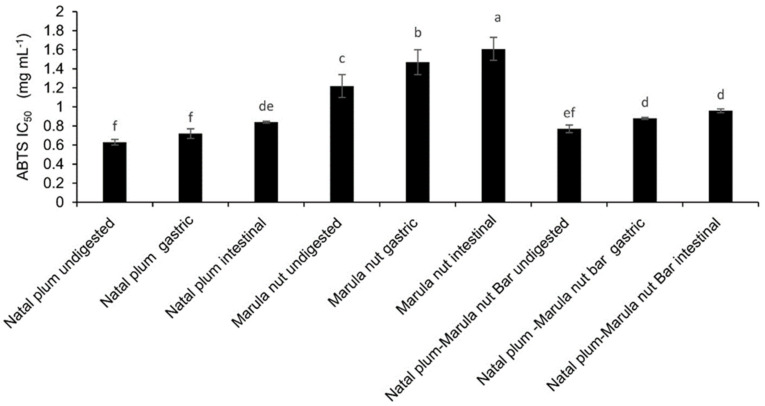
Effect of co-ingestion of Natal plum and Marula nut in a bar on the antioxidant capacity Means followed by the same letter in a bar are not significantly different at *p* < 0.05.

The ABTS radical scavenging capacity also followed the following order Natal plum > Natal Plum–Marula nut bar > Marula nuts (Figure 4). Simulated gastrointestinal digestion of Marula nuts significantly reduced their ABTS antioxidant capacity. However, only intestinal digestion significantly reduced the ABTS radical scavenging capacity of Natal plums. This may be because of the degradation of phenolic compounds that have higher ABTS antioxidant capacity in the simulated intestinal phase (pH neutral to alkaline), whilst they may be stable in the gastric phase. A decrease in ABTS antioxidant capacity has also been reported when apples were digested [31] or when persimmon fruit flours were digested [32]. The ABTS antioxidant capacity of the digested Natal plums after the intestinal stage was not significantly differ to that of the bar throughout digestion. Although the simulated digestion of the bar significantly reduced the ABTS antioxidant capacity after the gastric phase, the intestinal digestion did not significantly affect the ABTS antioxidant capacity. This may be due to a protective effect on phenolic compounds which have high ABTS activity or the deprotonation of the hydroxyl groups on phenolic compounds [33].

**Figure 5:**
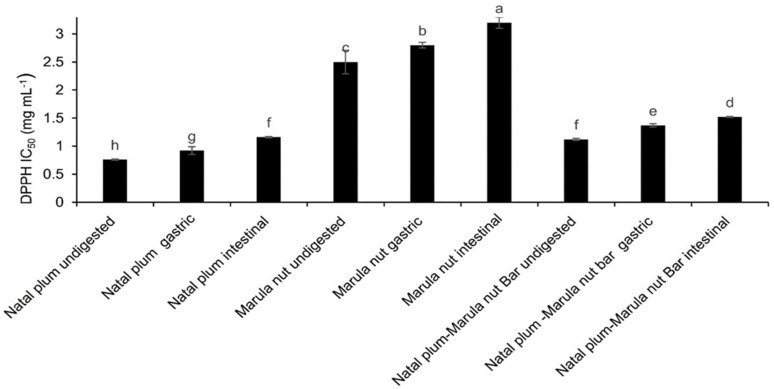
Effect of co-ingestion of Natal plum and Marula nut in a bar on the antioxidant scavenging activity. Means followed by the same letter in a bar are not significantly different at *p* < 0.05.

The DPPH radical scavenging capacity of Natal plum was the highest, whilst that of Marula nuts was the lowest (Figure 5). Combining Natal plums and Marula nuts in the bar reduced the DPPH capacity of the Natal plums because of the low DPPH antioxidant capacity of the nuts. Furthermore, the DPPH antioxidant capacity decreased with digestion for Natal plums, Marula nuts and the bar. This may be due to the decrease in the TPC possibly owing to degradation of phenolics to other products which have a lower DPPH antioxidant capacity. A strong positive relationship has been observed between TPC and DPPH when fruit seeds were digested suggesting that phenolic compounds were responsible for the DPPH activity [29]. Furthermore, antioxidants may be more reactive at acidic pH in the gastric phase and less reactive at near neutral to alkaline pH in the intestinal phase due to racemisation at higher pH, resulting in a decrease in antioxidant activity [34].

### 2.5. Correlations between Total Phenolics or Antioxidant Capacity with Different Phenolic Compounds

**Figure 6:**
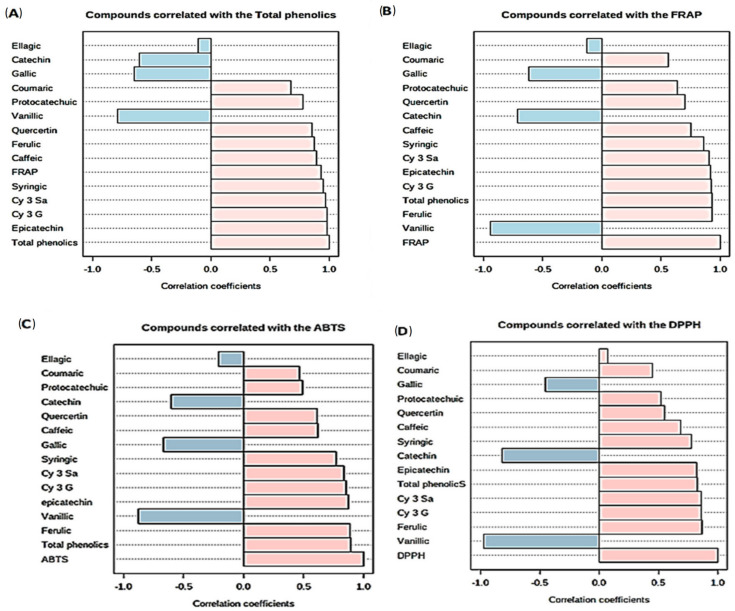
(**A**–**D**) Correlation of total phenolic content (**A**), FRAP antioxidant capacity (**B**), ABTS (**C**) and DPPH (**D**) radical scavenging capacities with phenolic compounds.

There was a strong positive correlation between TPC and FRAP (r = 0.93), epicatechin (r = 0.98), syringic acid (0.95), ferulic acid (0.88), Cy-3-Sa (0.97) and Cy-3-G (0.98), which was significant at *p* < 0.01. A strong positive correlation between FRAP and TPC or TPC and anthocyanins has been observed previously as stated by Seke et al. [5]. There was also a similar observation for FRAP which correlated positively with all phenolic compounds save for vanillic acid, gallic acid, catechin and ellagic acid, although the correlation with protochatechuic acid and coumaric acid was not significant at *p* < 0.05. The ABTS radical scavenging also showed a strong positive correlation with TPC (0.89), ferulic acid (0.88), epicatechin (0.87), Cy-3-Sa (0.84), Cy-3-G (0.86) and syringic acid (0.77) whilst DPPH radical scavenging was positively correlated to ferulic acid (0.87), Cy-3-Sa (0.86), Cy-3-G (0.86), TPC (0.83) and epicatechin (0.82) at *p* < 0.01. This correlation is as previously observed by Seke et al. [5,6] and shows that the delayed degradation of anthocyanins and increase in phenolic bioaccessibility is important to preserve antioxidant capacity. All correlations are shown in Figure 6A–D.

### 2.6. Effect of Co-Ingestion on α-Glucosidase Inhibition

**Figure 7:**
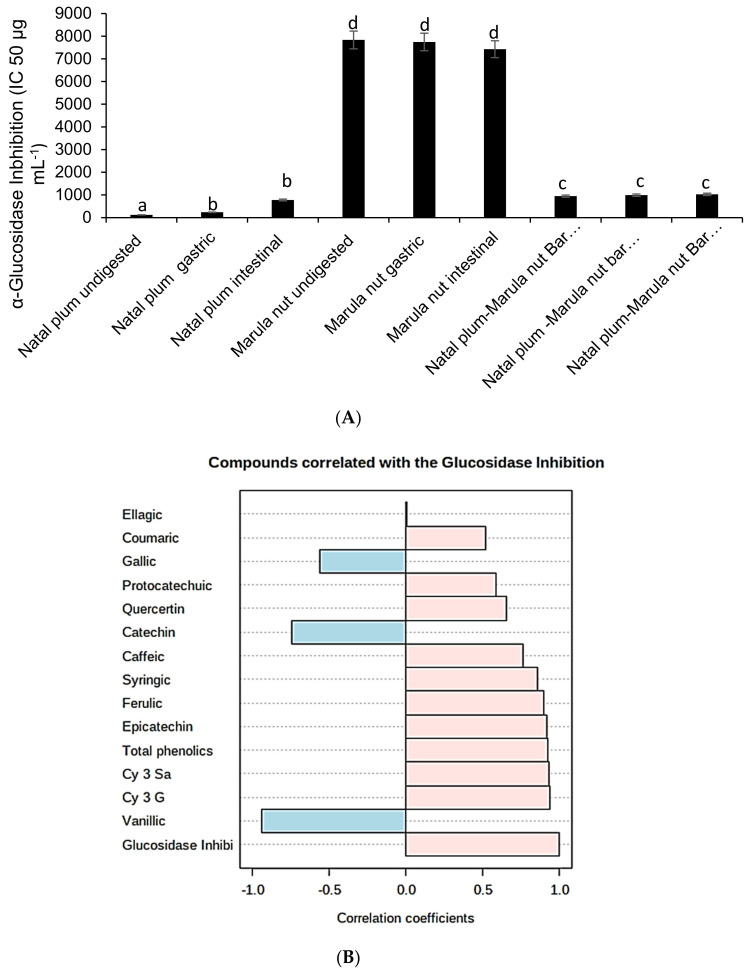
(**A**) Effect of simulated digestion on α-glucosidase inhibition capacity of Natal plums, Marula nuts and the bar. (**B**) Correlation of α-glucosidase inhibition capacity with phenolic compounds.

The order of α-glucosidase inhibition capacity was Natal plum > Natal Plum–Marula nut bar > Marula nuts (Figure 7A). Combining Natal plums and Marula nuts in the bar significantly (*p* < 0.05) reduced the α-glucosidase inhibition capacity due to the low inhibition capacity of Marula nuts. Simulated digestion significantly (*p* < 0.05) reduced the α-glucosidase inhibition capacity of Natal plums and Marula nuts but did not affect the Natal plum–Marula nut bar. This may be due to the protective effects of co-ingesting the Marula nuts with the Natal plum. There was a strong positive correlation between the Cy-3-Sa (r = 0.93) or Cy-3-G (0.94) and the α-glucosidase inhibition capacity at *p* < 0.0001 (Figure 7B). The higher bioaccessibility of the anthocyanins may have resulted in this observation. Anthocyanins Cy-3-G has been reported to have antidiabetic activity [26]. Treatment of C2C12 myotubes with conditioned media from Cy3G-treated adipocytes resulted in an increase in metabolism-related gene expression. Matsukawa et al. [35] showed by modulating adipocyte differentiation and insulin sensitivity, black soybean and its bioactive compound Cy3G may be used to prevent or alleviate type 2 diabetes. Similarly, Cy-3-Sa from Andean elderberry (*Sambucus nigra* L.) showed potential antidiabetic activity [36]. Hyperglycemia was corrected by the polar extract, while insulin secretion was lowered by the lipophilic extract [36], hence preventing of degradation of anthocyanins preserved the α-glucosidase inhibition capacity.

## 3. Materials and Methods

### 3.1. Materials

At the red colour maturity stage, Natal plums were harvested from the gardens of Tshwane University of Technology in Pretoria, Gauteng, South Africa. Fruits were washed, deseeded, and stored at −80° after harvesting. Kernels of Marula nut were obtained from Phalaborwa, Limpopo province from the rural framers and crushed with a stone the nut from the shell. Pancreatin from porcine pancreas, pepsin from porcine stomach mucosa, phenolic standards were purchased from Sigma Aldrich, Johannesburg, South Africa.

### 3.2. Bar Preparation

Natal plum was pureed using a blender and mixed with Marula nuts in a ratio 1:1 (*v*/*v*). The mixture was poured in a lined tray and dehydrated at 50 °C for 72 h in a conventional oven were stored under −80° until further analysis.

### 3.3. Simulated Gastro-Intestinal Digestion

#### 3.3.1. Preparation of Materials Simulated Digestion Fluid and Digestion Procedure

Simulated digestion was carried out following the method described by Brodkorb et al. [37] and Seke et al. [5]

*Oral phase*: A total of 5 g of the Natal plum fruit or Marula nut or Natal plum bar was weighed into a volumetric flask and 10 mL of SSF containing 75 U mL^−1^ amylase was added. The mixture was agitated at 170 rpm for 2 min at 37 °C.

*Simulated gastric digestion*: A 20 mL simulated gastric fluid was added to the bolus and the pH was adjusted to 2.5. The pepsin solution (2000 U mL^−1^ in 0.1 M HCl, pH 2.2) was added to initiate simulated gastric digestion. The mixture was incubated under agitation at 170 rpm for 2 h at 37 °C to mimic the gastric phase; a 10 mL sample was collected and cooled on ice for 10 min to stop reactions and stored at −80 °C for further observation.

*Simulated small intestinal digestion:* To the remaining mixture, 20 mL of simulated small intestinal fluid was added, and the pH adjusted to 7.5. Thereafter, 1.75 mL of a pancreatin solution (800 U mL^−1^), 20 mg of bovine bile extract, 20 mg of porcine bile extract, and 14 μL of 0.3 M CaCl_2_ were added, and the mixture was maintained under agitation at 37 °C for 2 h. The digestion ended by cooling the samples in an ice bath and immediately storing the sample at −80 °C for further analysis. A digestion blank containing enzymes and simulated digestion fluids was also kept. Table 2 shows the composition of simulated salivary fluid, simulated gastric fluid and simulated intestinal fluid.

**Table 2:** Composition of Simulated salivary fluid (SSF), Simulated gastric fluid (SGF) and Simulated intestinal fluid (SIF).

Stock Solutions	SSF	SGF	SIF
Distilled water	400 mL	400 mL	400 mL
2 M NaCl	-	11.8 mL	9.6 mL
0.5 M KCl	15mL	6.9 mL	6.8 mL
1 M NaHCO_3_	6.8 mL	12.5 mL	42.5 mL
0.3 M CaCl_2_·H_2_O	0.025mL	0.005 mL	0.04 mL
0.5 M KH_2_PO_4_	3.7 mL	0.9 mL	0.8 mL
0.5 M (NH_4_)_2_CO_3_	0.06mL	0.5 mL	-
0.15 M MgCl_2_·H_2_O	0.5 mL	0.4 mL	1.1 mL
6 M HCl	0.09mL	0.7 mL	0.7 mL
pH	6.8 ± 0.2	1.30 ± 0.02	8.1 ± 0.2

#### 3.3.2. Extraction of Phenolics from Undigested and Digested Natal Plum Fruit, Marula Nut or Natal Plum–Marula Nut Bar Samples

Sample extracts were produced using a previously described method, with slight modifications [5]. Natal plum, Marula nuts or bar samples (100 mg) were defatted with hexane and dried. Thereafter, extraction was done using 80:20 methanol/water (10 mL) and was ultrasonicated for 30 min at 30 °C then centrifuged (Hermle Z326k, Hermle Labortechnik GmbH, Wehingen, Germany) at 3000× *g* for 20 min at 4 °C. Extractions were performed in triplicate, and the extracts were used for the analysis of the total phenolic content (TPC), antioxidant capacity, antidiabetic activity, phenolic profile using UPLC/QTOF/MS.

### 3.4. Determination of Total Phenolic Content (TPC)

The determination of TPC was according to a method which was originally developed by Singleton et al. [38] and Seke et al. [5] using Folin-Ciocalteu’s phenol reagent. To 0.5 mL of diluted extract (100 μg mL^−1^), 2.5 mL of Folin–Ciocalteu reagent (diluted 10 times with water), and 2 mL of Na_2_CO_3_ (7.5%) were added. The absorbance of the mixture was measured at 750 nm using a 94-well multiplate reader ((BMG LABTECH GmbH, SpectroStar Nano, Ortenberg, Germany) and the TPC results were expressed as mg gallic acid equivalent 100 g^−1^ fresh weight basis.

### 3.5. Antioxidant Activity

#### 3.5.1. DPPH Radical Scavenging Activity

The 2,2-diphenyl-1-picrylhydrazyl (DPPH) activity was determined by following the methodology described by Seke et al. [4]. Different concentrations of each sample (50 µL) were mixed with 200 µL of DPPH solution (0.02%. *w*/*v*) and incubated in the dark for 1 h. The decolourisation of DPPH• was measured at 517 nm using a 94-well multiplate reader. Analyses were performed in triplicate and the results were expressed as IC_50_ µg mL.

#### 3.5.2. Ferric-Reducing Antioxidant Power (FRAP)

The Ferric-reducing power was evaluated as described by Huang et al. [39]. Briefly, FRAP was determined based on the reduction of Fe3^+^-TPTZ to a blue coloured Fe^2+^-TPTZ. The FRAP reagent was prepared by mixing 300 mM acetate buffer (pH 3.6), 10 mM TPTZ and 20 mM FeCl_3_·6H_2_O in a ratio of 10:1:1. A total of 200 µL of the sample (different concentrations) was mixed with 50 µL of the FRAP reagent. and the absorbance was measured at 700 nm using a 94-well multiplate reader.

#### 3.5.3. ABTS Radical Scavenging Capacity

The 2,2-azinobis-3-ethylbenzothiazoline-6-sulfonic acid (ABTS) radical scavenging capacity was determined according to David et al. [40]. ABTS (7 mM) was dissolved in potassium persulfate (2.45 mM) and kept in the dark for 12 h. to produce the radical solution (ABTS^•+^). The radical solution. it was diluted with phosphate buffer saline (PBS 50 mM. pH 7.4). until reaching an absorbance of 0.7 at 734 nm. A total of 10 µL of each sample was mixed with 200 µL of ABTS solution. incubated for 6 min. and its absorbance measured at 734 nm using a 94-well multiplate reader. The results were expressed as IC_50_ µg mL^−1^.

### 3.6. Identification of Anthocyanins in Natal Plum Using UHPLC/Q-TOF-MS

The anthocyanins in Natal plum were identified using a method previously reported by Seke et al. [5] using a Waters ultra-performance liquid chromatograph (UPLC) (with a Water Acquity photodiode array detector (PDA), coupled to a Synapt G2 quadrupole time-of-flight (QTOF) mass spectrometer (MS) (Waters, Milford, MA, USA). The anthocyanins were identified after using an electrospray ion source operating with a cone voltage of 15 V, desolvation temperature of 275 °C and a desolvation gas flow at 650 L h^−1^ in negative ESI mode. The mobile phases A was milliQ water containing 10% formic acid, and mobile phase B was acetonitrile containing 10% formic acid. The gradient was: 0–1.5 min, 0.5% B; 1.5–4 min, 0.5–11% B; 4–23 min, 11–15% B; 23–28 min, 15–100% B; 28–38 min, 100% B; 38–39 min, 100–0.5% B; 39–49 min, 0.5% B as reported by Ndou et al. (2019). Flow rate was set at 0.5 mL/min and column temperature was set at 50 °C. The anthocyanins and other phenolic compounds were quantified using standards and expressed as mg kg^−1^. Supplementary Table S1 presents the regression equation, correlation coefficient (R^2^), limit of detection (LOD), limit of quantitation (LOQ) of phenolic compounds by UHPLC/Q-TOF-MS.

### 3.7. Bioaccessibility Calculation

The bioaccessibility (%) after digestion of a particular compound is determined by comparing the initial content of the compound in the undigested sample to the final content left after digestion and is calculated using Equation (1):

(1) $$\mathrm{Digestive}\;\mathrm{bioaccessibility}\;(\%)=(\frac{B_{AD}}{B_{BD}})\times 100$$

where *B_AD_* is the amount obtained after the simulated digestion and *B_BD_* is the total initial content of the compound determined in the undigested sample [41].

### 3.8. Determination of α-Glucosidase Inhibition

The in vitro α-glucosidase inhibitory activity of the sample extracts was determined following a method outlined by Apostolidis & Lee [42], with some minor modifications. Briefly, a mixture of 50 µL of the sample and 100 µL of 0.1 M phosphate buffer (pH 6.9) containing the glucosidase solution (1 U mL^−1^) was incubated in 96-well plates at 25 °C for 10 min. Thereafter, 50 µL of 5 mM, p-nitrophenyl-α-d-glucopyranoside solution in 0.1 M phosphate buffer (pH 6.9) was added to each well and further incubated at 25 °C for 5 min. A microplate reader was used to determine the absorbance of the sample, blank and control at 405 nm a 94-well multiplate reader. Analyses were performed in triplicate and the results were expressed as IC_50_ µg mL^−1^.

### 3.9. Data Analysis

All analyses were done in triplicate. Data were expressed as mean ± standard deviation. Statistical analysis was done by one-way analysis of variance (ANOVA) and Tukey’s test for comparison of means (*p* < 0.05) using Minitab (version 9.0. Minitab Inc., State College, PA, USA). Linear regression was used to determine the correlation described by the Pearson’s correlation coefficient (r) using Metaboanalyst v5.0.

## 4. Conclusions

Co-ingestion of Natal plums and Marula nuts in a fruit bar increases the bioaccessibility of Cy-3-Sa or Cy-3-G and phenolic compounds, preserves antioxidant capacity, and increases α-glucosidase inhibition after simulated digestion compared to ingesting the fruit and nut separately in this study. In fact, co-ingestion led to increased intestinal stability and bioavailability of the Cy-3-Sa or Cy-3-G. Therefore, the Marula nut food matrix could have increased the bioavailability of anthocyanin-based cyanidins. The produced bar is a potential functional snack. The growing popularity of fruit and nut bars as convenient breakfast foods demands that food processors gain a deep understanding of the interactions between their ingredients. However, further studies should be carried out to determine the proximate analysis, quality and the sensory properties of the Natal-plum fruit bar.

## Figures and Tables

**Table 1:** Changes in bioaccessibility of anthocyanins and other polyphenols after simulated co-digestion of Natal plum and Marula nut in a bar on fresh weight basis.

Phenolic Compound (mg/kg)	Natal Plum Undigested	Natal Plum Gastric Phase	Natal Plum Intestinal Phase	Bio Accessibility (%)	Marula Undigested	Marula Gastric	Marula Intestinal	Bio Accessibility (%)	Natal Plum–Marula Bar Undigested	Natal Plum–Marula Gastric Phase	Natal Plum–Marula Intestinal Phase	Bio Accessibility (%)
Gallic	1.0 ± 0.0e	0.5 ± 0.0e	2.3 ± 0.4e	230	7.9 ± 0.7c	5.1 ± 0.2d	33.7 ± 0.1a	426.6	8.5 ± 1.1c	2.3 ± 0.1e	15.0 ± 0.1b	176.5
Protocatechuic	39.9 ± 3.9a	18.2 ± 0.1c	22.9 ± 3.6b	57.4	13.4 ± 1.5d	11.6 ± 1.0de	9.3 ± 0.3e	69.4	5.6 ± 1.4f	4.2 ± 0.8f	7.5 ± 0.3ef	133.9
Cy-3-G	267.9 ± 12.6a	175.7 ± 15.3b	87.9 ± 5.4c	32.8	0.0	0.0	0.0		88.3 ± 6.7c	74.8 ± 1.9c	63.5 ± 8.8c	71.9
Cy-3-Sa	422.1 ± 13.8a	384.3 ± 12.3b	167.6 ± 7.6c	39.7	0.0	0.0	0.0		125.8 ± 11.1s	116.9 ± 1.0d	100.9 ± 9.1d	80.2
Catechin	126.3 ± 11.9c	60.5 ± 1.1d	90.7 ± 4.5d	71.8	229.7 ± 3.9a	227.0 ± 2.2a	189.8 ± 2.3b	82.6	227.8 ± 8.7a	147.0 ± 3.0c	120.7 ± 4.5c	52.8
Epicatechin	5.0 ± 0.1a	3.3 ± 0.2b	2.1 ± 0.1bcd	42	1.0 ± 0.0cd	0.8 ± 0.1d	0.6 ± 0.0d	60	2.4 ± 0.2bc	1.6 ± 0.2cd	1.4 ± 0.1cd	58.3
Caffeic acid	28.9 ± 1.4a	19.4 ± 0.5b	10.5 ± 1.2c	36.3	4.8 ± 1.1e	3.7 ± 0.2e	7.7 ± 0.0d	160.4	4.4 ± 0.2e	3.9 ± 0.2w	4.8 ± 0.3e	109.1
Vanillic acid	0.0	0.0 ± 0.0	0.0		18.5 ± 1.2a	17.3 ± 0.4a	14.6 ± 0.2b	78.9	7.8 ± 0.1c	7.8 ± 0.3c	5.9 ± 0.2c	75.6
Syringic acid	25.8 ± 1.7a	12.1 ± 0.6b	6.8 ± 0.7c	26.4	0.0	0.0	0.0		5.2 ± 0.6c	3.8 ± 0.2d	2.7 ± 0.1d	51.9
Ellagic acid	7.1 ± 0.9d	12.4 ± 0.2b	2.9 ± 0.2f	40.8	4.5 ± 0.3ef	4.9 ± 0.2e	16.0 ± 1.5a	355.5	6.3 ± 0.2d	7.1 ± 0.2d	9.8 ± 0.4c	155.6
Coumaric acid	55.5 ± 4.3a	17.4 ± 1.4d	24.0 ± 0.9b	43.2	24.0 ± 1.5b	17.1 ± 0.4d	20.5 ± 0.2c	85.4	23.1 ± 2.4b	12.9 ± 0.3e	22.0 ± 1.2bc	95.2
Ferulic acid	30.4 ± 1.5a	15.0 ± 0.9c	19.5 ± 2.1b	64.1	5.7 ± 0.5e	3.6 ± 0.2f	3.0 ± 0.1f	52.6	16.8 ± 1.8c	9.3 ± 0.9d	15.0 ± 0.9c	89.3
Quercetin	53.8 ± 2.2a	28.2 ± 0.8b	28.7 ± 1.1b	53.3	25.7 ± 1.2b	16.2 ± 0.4c	9.1 ± 0.2d	35.4	15.2 ± 2.2c	11.4 ± 1.8cd	15.0 ± 0.4c	98.7
Total poly phenols	1063.7	747	465.9		335.2	307.3	304.3		537.2	403	384.2	

Values means ± SD. Bioaccessibility (BA). Values in the same row with different letters are significantly different at *p* < 0.05.

## Data Availability

The data presented in this study are available in the present article and Supplementary files.

## Supplementary Materials

The following are available online. Table S1: Regression equation, correlation coefficient (R^2^), limit of detection (LOD), limit of quantitation (LOQ) of phenolic compounds by UHPLC/Q-TOF-MS, Table S2: Identification and quantification of anthocyanins using UPLC/QTOF/MS.
