# Supplementary material for: Co-Ingestion of Natal Plums (Carissa macrocarpa) and Marula Nuts (Sclerocarya birrea) in a Snack Bar and Its Effect on Phenolic Compounds and Bioactivities
Source: Molecules. 2022 Jan 4;27(1):310. doi: 10.3390/molecules27010310 (PMC8746984; doi:10.3390/molecules27010310)
Supplement: Supplementary file 1 [file molecules-27-00310-s001.zip › molecules-1478426-supplementary.pdf]

Supplementaru Material

Co-ingestion of Natal Plums (*Carissa macrocarpa*) and Marula Nuts (*Sclerocarya birrea*) in a Snack Bar and Its Effect on Phenolic Compounds and Bioactivities

Vimbainashe E. Manhivi [1] Retha M. Slabbert [2] and Dharini Sivakumar [1,*]

[1]Phytochemical Food Network Group, Department of Crop Sciences, Tshwane University of Technology, Pretoria West 0001, South Africa; ManhiviVE@tut.ac.za
[2] Department of Horticulture, Tshwane University of Technology, Pretoria West 0001, South Africa; SlabbertMM@tut.ac.zam
* Correspondence: SivakumarD@tut.ac.za

Table S1. Regression equation, correlation coefficient (R^2), limit of detection (LOD), limit of quantitation (LOQ) of phenolic compounds by UHPLC/Q-TOF-MS.

Phenolic compounds	Retention time(min)	Regression equation	R^2	LOD (µg/mL)	LOQ (µg/mL)
Gallic acid	10.197	y =99324x+55626	0.998	0.05	0.19
Protochatechuicacid	10.855	y=19722x-341718	0.998	3.2	10.9
Catechin	11.84	y=2828x-69172	0.999	3.2	15.7
Epicatechin	14.2	y=35316x+193517	0.997	1.4	3.3
Caffeic acid	14.509	y=28189x-161653	0.999	2.2	7.2
Chlorogenic acid	14.724	y=71930x-2110,5	0.995	0.11	0.37
Quercetin	16.77	y=12091x-170181	0.996	7.7	25.8
Ferulic acid	17. 93	y=20067x-279209	0.999	3.3	11.9
p-Coumaric acid	17.54	y=6925.3x-62646	0.999	10.25	34.17
Kaempferol	16.448	y=26658x+492185	0.996	2.7	9.3
Syringic acid	15.44	y=75813x-107617	0.999	0.05	0.18
Ellagic acid	15. 996	y=20110x+9484.9	0.997	0.31	1.2
Cyanidin-3- sambubioside		y = 1.958x +1.619	0.99	0.12	0.47
Cyanidin-3-*O*- glucoside content		y = 1.825x + 0.538	0.99	0.03	0.25

Table S2. Identification and quantification of anthocyanins using UPLC/QTOF/MS.

Average Rt(min)	Molecular Formula	Expected Mass m/z	Observed Average m/z	Tentative Compound Name
13.43	$C_{26}H_{29}O_{15}$	581.5	579.12872	Cyanidin 3-O-Sambubioside
15.822	$C_{21}H_{22}O_{11}$	449.1083	449.10825	Cyanidin 3-O-Glucoside
